# ECG Changes in 8-Year-Old Boy with Pulmonary Edema after Head Injury

**DOI:** 10.1100/tsw.2006.108

**Published:** 2006-05-17

**Authors:** Bojko Bjelakovic, Vladislav Vukomanovic, Ljiljana Saranac, Ivan Stefanovic

**Affiliations:** ^1^ Clinic of Pediatrics and Clinic of Neurosurgery, Clinical Center, Nis and Mother and Child Care Institute of Serbia, Serbia; ^2^ Department of Paediatric Cardiology, Mother and Child Health Institute, Belgrade, Serbia

**Keywords:** CNS, pulmonary, edema, ECG, myocard, Yugoslavia

## Abstract

This is a case story of an 8-year-old boy with no prior history of cardiac disease who developed acute pulmonary edema with ECG changes similar to transmural myocardial infarction after basilar skull fracture. Biochemical evaluation showed elevated total creatine kinase activity –1,350 U/L with 12% MB isoenzyme fraction. The brain scan on admission showed cerebral edema with ethmoidal sinuses hemorrhage. Neurogenic pulmonary edema following CNS damage is an extremely rare entity in the pediatric population and there are few reports. There are many proposed mechanisms and explanations of its origin. Based on previous reports and experimental studies, the cause of “neurogenic” pulmonary edema may be of cardiac as well as of noncardiac origin.

## INTRODUCTION

Pulmonary edema following CNS damage is often called neurogenic pulmonary edema. Such an entity is extremely rare in the pediatric population and there are few reports[1]. Subarachnoid hemorrhage, massive head injuries, and stroke are the frequent causes of pulmonary edema, but the precise pathogenesis and the explanations for neurogenic pulmonary edema have not been defined[2].

## CASE REPORT

An 8-year-old boy was admitted to our medical center on May 4th, 2004 due to head injury and loss of consciousness. Half an hour before, he had stood below a goal post, which accidentally fell on his head. On admission to the emergency room, the patient was comatose, cyanotic, with small unresponsive pupils, afebrile, hypotensive TA = 90/60, SF = 100/min, with Cheyne-Stokes breathing, weak and irregular pulse. On physical examination, there were large, left frontoparietal succedaneum swelling and marked CSF (cerebrospinal fluid) left othoraghia. Pulmonary auscultation revealed moist, bubbly crackles in the lower and middle portions of both lungs and cardiovascular auscultation revealed muffled heart sounds.

Chest roentgenograms showed a normal-sized cardiac shadow; bilateral, fluffy, alveolar infiltrates (butterfly distribution); and no other signs of chest trauma. Tracheal suction produced frothy, pink-tinged secretion typical of pulmonary edema. A CT scan of the head performed 1 h after admission to hospital showed a many-sided fracture: left frontoparietal and occipital in left pyramidal area. There were also marked, generalized edema cerebri, trunci cerebri, and ethmoidal sinuses hemorrhage. Initial arterial blood gas analysis showed mixed respiratory and metabolic acidosis, pH = 7.20, pCO2 = 55 mmHg, pO2 = 62.7 mmHg, BE = –6.5, HCO3 = 15.2, O2 sat = 61%. Because of the increasing respiratory distress, the patient was intubated and hyperventilated with the fraction of inspired oxygen of FiO2 = 50%. The patient also received intravenous (i.v.) dexamethasone sodium phosphate in doses of 4 mg every 6 h, furosemide 1 mg/kg, and osmotherapy with i.v. mannitol 20% in a loading dose of 1.5 g/kg. During the initial evaluation, an i.v. line was established and blood was obtained for a complete blood count, electrolytes, glucose, creatinine, blood gases, and liver function. We also performed the first ECG 6 h after admission, which showed diffuse, flattened T wave and prolonged QT interval (see Figure 1).

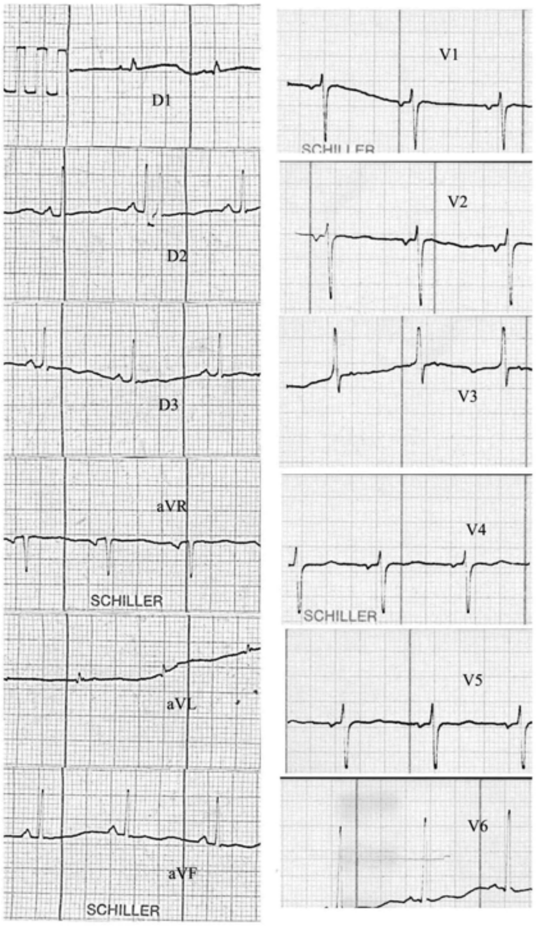

**FIGURE 1**. Diffuse, flattened T wave and prolongation of QT interval. Amputate (low) voltage R wave in leads V4, V5. Pathologic terminal forces of P wave in lead V1.

The next day, the condition did not change significantly. He was comatose, afebrile, mechanically ventilated, hypotensive TA = 90/50, with SF = 100/min. The arterial blood gases showed pH = 7.31, pCO2 = 30.6, pO2 = 82, BE = –2.5, HCO3 = 18.2, O2 sat = 83%. The second recorded ECG revealed flattened T wave in all leads, pathological Q waves in leads D1 and aVL, and amputate (low voltage) R wave in leads V2-V4 (see Figure 2). Because of the registrated ECG changes, we also controlled isoenzyme

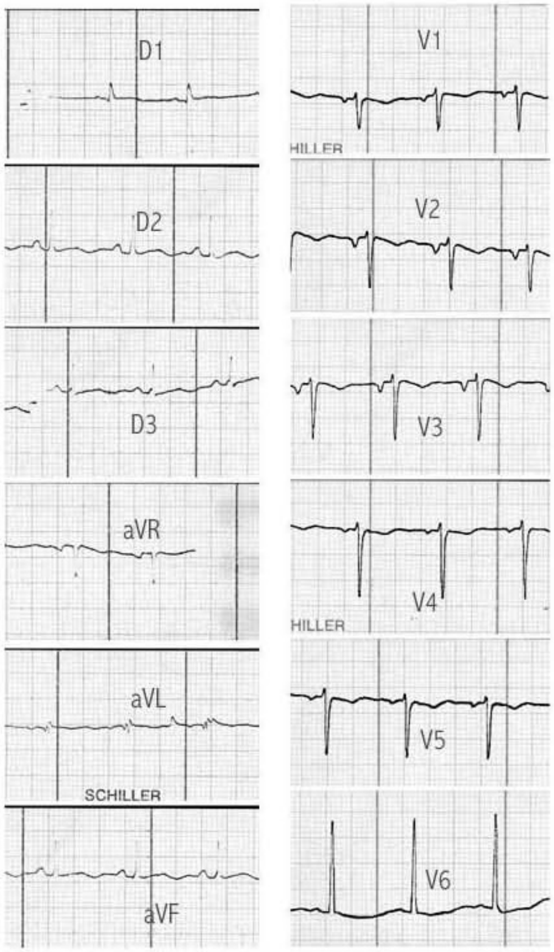

**FIGURE 2.** Flattened T wave in all leads. Pathological Q waves in leads D1 and aVL and amputate (low voltage) R wave in leads V2-V4. Pathological terminal forces of P wave in lead V1-V3.

fraction of creatine kinase activity CPK-MB, which was elevated with the maximum activity 33 h after the hospitalization: CPK1-MB = 162 U/L (12%) MB isoenzyme fraction.

On the third day, the condition did not improve despite a continuous therapy and the third ECG revealed the pathological Q waves in leads D1 and aVL, QS configuration in leads V2-V4, and diffuse, flattened T wave in all leads (see Figure 3). The value of CPK-MB was CPK2 - MB = 88 U/L (6.5%) MB isoenzyme fraction. The condition lasted 2 weeks without significant changes.

All the time during the hospitalization, the patient was deeply comatose, Glasgow Coma Scale 7 with various acid-base, coagulation, fluid, and electrolyte abnormalities, which were frequently corrected and controlled. On the 15th day, the patient was more alert with normal blood pressure (120/75) and other vital signs. On the 16th day, the mental status was markedly improved and he was definitively extubated. Finally, on the 20th day after admission, he was discharged to rehabilitation.

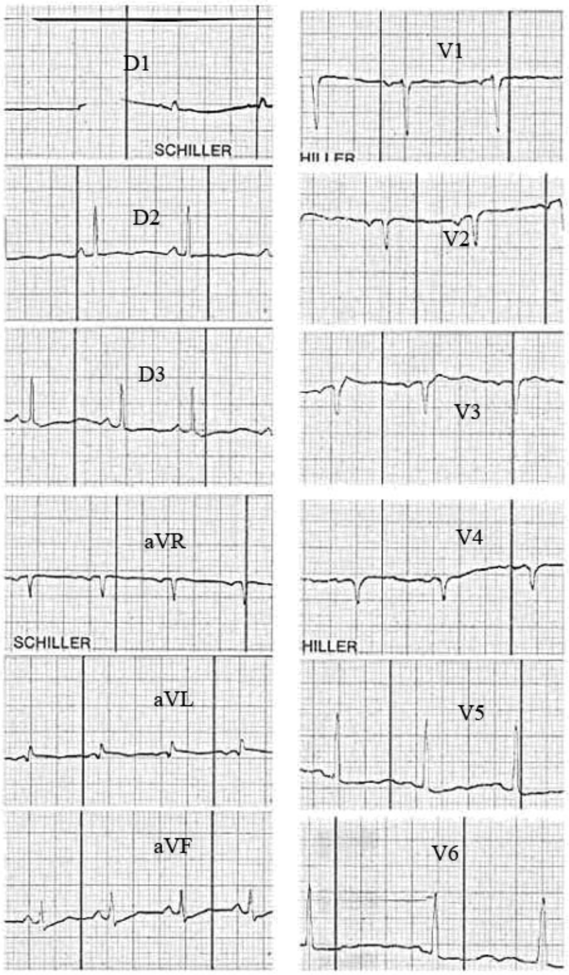

**FIGURE 3.** Pathological Q waves in leads D1 and aVL. QS configuration in leads V2-V4 and diffuse, flattened T waves in all leads.

## DISCUSSION

The most-mentioned noncardiac mechanisms of “neurogenic” pulmonary edema are based on massive autonomic discharge with catecholamine excess due to CNS derangement. As the consequence, rapid vasoconstriction with a shift of blood from the high-resistance systemic to the low-resistance pulmonary vascular bed, as well as rise in capillary endothelial permeability, are responsible for the onset of pulmonary edema. Experimental documentation of protein-rich pulmonary edema fluid in the face of relatively small changes in pulmonary hemodynamics support the hypothesis of increased capillary permeability in neurogenic pulmonary edema[3].

The rise in left atrial pressure secondary to systemic hypertension and myocardial dysfunction, due to catecholamine excess and myocitolisis, may also cause increased pulmonary capillary hydrostatic pressure[4]. In addition to echocardiography and other invasive methods revealing centrally mediated myocardial dysfunction, there are few biochemical parameters (markers of myocardial damage) proving this. Today, the rise in serums myoglobin and creatinin phosphokinase – myocardial fraction isoenzyme (CPK-MB) is well confirmed in CNS disorders[6,7]. Stopfkuchen found high serum CPK-MB values in children with isolated head injury[8]. Kaste et al. described significantly higher values of CPK-MB isoenzyme activity than CPK-brain fraction activity in patients with brain injury, coincident with ECG changes and pathoanathomic confirmation of myocardial damage[7].

Schell et al. reported a case of a patient with subarachnoid hemorrhage with echocardiographic (depressed left ventricular systolic function) and radionuclear abnormalities of left ventricular dysfunction together with elevated CK-MB isoenzyme as the cause of "cardiogenic" pulmonary edema. Despite these findings, they did not record ECG changes[9]. Hammermeister and Reichenbach reported a case of subarachnoid hemorrhage with pulmonary edema and electrocardiographic pattern consistent with subendocardial myocardial infarction[3].

We describe a patient with no previous heart disease who developed acute pulmonary edema as the consequence of cranial injury and following cerebral edema. A constellation of findings, including ECG changes similar to transmural anterolateral myocardial necrosis and elevated CK-MB isoenzyme activity on the second and third day of hospitalization, pointed to a direct myocardial injury leading to cardiac failure. Parallel to the ECG signs of anterolateral myocardial infarction on the second and third day of hospitalization, pathologic Q wave in leads D1 and aVL, and amputated R wave (later QS wave) in leads V2-V4, we also noticed pathologic terminal forces of P wave in lead V1 (PTF-V1) PTF-V1 > 0.4 mmsec, indicating left atrial enlargement or high diastolic pressure[5]. This coincided with the presence of hypotension on physical examination, which also confirmed the hypothesis of myocardial dysfunction as the cause of cardiac failure and pulmonary edema[4].

Although, there are no data in clinical studies that confirm the role of cerebral edema in pathogenesis of myocardial damage, Weir (in his retrospective clinicopathological analysis of 78 cases with fatal subarachnoid hemorrhage) found significantly higher incidence of cerebral edema in the group with pathological pulmonary edema than in the group without. He also found that patients with pulmonary edema tended to be younger and died more quickly, but he could not confirm this statistically ($p > 0.05$)[10]. In addition, Rosenbloom described massive ST elevation without myocardial injury in a patient with fulminate hepatic failure and cerebral edema[11].

The evidence of ECG changes with the absence of classical triads (ischemia, lesion, and necrosis) also pointed to a direct myocardial injury due to CNS derangements as the cause of pulmonary edema. Zelikovic et al. reported a child with precordial pain and electrocardiographic abnormalities simulating myocardial infarction associated with an increased intracranial pressure[12]. Although there are many different ECG signs of CNS influences on myocardium, these ECG changes can be useful for every clinician in similar clinical cases. Since the differentiation between "cardiogenic" and "noncardiogenic" mechanisms of pulmonary edema has therapeutic implications, the role of ECG recording in establishing the cause is stressed[13].

## References

1. Kirkpatrick, M., Engelmann, H., and Minns, A. (1989) Symptoms and signs of progressive hydrocephalus. *Arch. Dis. Child*. **64**, 124–128.
2. Fein, I.A. and Rackow, E.C. (1982) Neurogenic pulmonary edema. *Chest* **81**, 318–320,
3. Hammermeister, K.E. and Reichenbach, D.D. (1969) QRS changes, pulmonary edema, and myocardial necrosis associated with subarachnoid hemorrhage. *Am. Heart J*. **78(1),** 94–100.
4. Tamaki, T., Isayama, K., Yamamoto, Y., and Teramoto, A. (2004) Cardiopulmonary hemodynamic changes after severe head injury. *Br. J. Neurosurg*. **18(2),** 158–163.
5. Hopkins, C.B. and O'Neill, B. (1989) Electrocardiographic diagnosis of left atrial enlargement. *J. Electrocardiol*. **22,** 4.
6. Sato, Y., Mizoguchi, K., Sato, Y., Hayashi, T., Shojima, K., Okamoto, Y., and Kayi, M. (1985) Serum myoglobin an acute cerebrovascular diseases. *Kurume Med. J.* **32,** 203–207.
7. Kaste, M., Juha, H., Hannu, S., Matti, H., and Aarne, K. (1981) Creatine kinase isoenzymes in acute brain injury. *J. Neurosurg*. 511–515.
8. Stopfkuchen, H., Schranz, D., Saizmann, G., Jungst, B.K., Wiss, M., and Schwarz, M. (1984) Diagnostic value of CK-MB isoenzyme determination in children with craniocerebral injuries and polytrauma. *Monatsschr. Kinderheilkd*. **132(8),** 594–599. [German]

9. Schell, A.R., Mohandas, M.S., Sandor, A.F., and Anilkumar, R.P. (1987) Pulmonary edema associated with subarachnoid hemorrhage. *Arch. Intern. Med*. **147,** 591–592.
10. Weir, K.B. (1978) Pulmonary edema following fatal aneurism rupture. *J. Neurosurg*. **49(4),** 502–507.
11. Rosenbloom, A.J. (1991) Massive ST elevation without myocardial injury in patient with fulminant hepatic failure and cerebral edema. *Chest* **100(3),** 870–872.
12. Zelikovic, I.N., Kaplan, C.B., Varsano, I.B., Aygen, M.M., and Shalit, M.J. (1981) Precordial pain and electrocardiographic abnormalities simulating myocardial infarction associated with increased intracranial pressure in child. *Helv. Pediatr. Acta* **36(5),** 483–487.
13. Rogers, M.C., Zakha, K.G., Nugent, S.K., Gioa, F.R., and Epple, L. (1980) Electrocardiographic abnormalities in infants and children with neurologic injure. *Crit. Care Med.* **8**, 213–214.

## BIOSKETCHES

**Bojko Bjelakovic**, **MD**, **MSc,** Clinic of Pediatrics, Clinical Center, Nis, Serbia, Yugoslavia. E-mail: bojko@medianis.net

**Vladislav Vukomanovic, M.D., Ph.D.,** Assistant professor, Head, Department of Paediatric Cardiology, Mother and Child Health Institute, Belgrade, Yugoslavia.

**Ljiljana Saranac**, **MD, PhD**, Associate Professor, Clinic of Pediatrics, Clinical Center, Nis, Serbia, Yugoslavia.

**Ivan Stefanovic**, **MD, PhD**, Professor, Clinic of Neurosurgery, Clinical Center, Nis, Serbia, Yugoslavia.
